# Posterior Mediastinal Mass: An Uncommon Presentation of a Branchial Cleft Cyst

**DOI:** 10.7759/cureus.30924

**Published:** 2022-10-31

**Authors:** Wendolin J Ortiz, Elva C Ahumada, Olivaldo L Paz-Moreno, Robert L McKowen, Mario Cervantes

**Affiliations:** 1 Pathology, HCA (Hospital Corporation of America) Houston Healthcare West, Houston, USA; 2 General Surgery, Universidad Autónoma de Baja California, Mexicali, MEX; 3 Pathology, HCA (Hospital Corporation of America) Houston Healthcare Pearland, Houston, USA; 4 Pathology, Universidad Xochicalco, Ensenada, MEX; 5 Pathology, Universidad Autónoma de Baja California, Tijuana, MEX; 6 Cardiothoracic Surgery, HCA (Hospital Corporation of America) Houston Healthcare West, Houston, USA

**Keywords:** anomaly, embryology, posterior mediastinum, branchial cleft cyst, mediastinal mass

## Abstract

Branchial cleft anomalies are a heterogeneous group of congenital disorders that theoretically emerge due to incomplete obliteration of the branchial apparatus, components of the six main pairs of pharyngeal arches, during embryonic development. They can result in a cyst, a sinus, or a fistula. For a congenital lateral neck mass, they represent the most common diagnosis in pediatric neck pathology. Its location is usually in the cervical area, anterior to the sternocleidomastoid muscle. In adults, they present with symptoms such as acute suppurative thyroiditis and recurrent cervical abscess. In this paper, we report the case of a 54-year-old Hispanic woman with a recent history of a left posterior mediastinal mass, detected on computed tomography (CT) scan while studying her recent onset of asthma. The patient underwent an assisted thoracoscopic excision. During the procedure, the mass appeared to be a very large cyst; on histopathological examination, the cyst was determined to be a branchial cleft cyst. To our knowledge, this is the first reported case of this entity located in the posterior mediastinum in an adult patient. Being an unusual and interesting case, it highlights the idea of considering these anomalies when establishing a differential diagnosis of a posterior mediastinal mass.

## Introduction

The temporary slit-like structures that appear between the fourth and seventh weeks of gestation on the lateral sides of the developing fetus resemble the gills (branchia in Greek) in fish and amphibians. They form the branchial apparatus, normal embryonic arches that should be absent once a fetus has fully developed. If these structures persist due to incomplete obliteration of the branchial apparatus, they may present as branchial cysts, fistulas, or sinuses [1,2].

According to the literature, there is no tendency toward gender or ethnic group for these anomalies; the true incidence in the United States is not known accurately due to the variety of anatomical presentations that complicate the reporting of cases. They are classified into the first, second, third, and fourth anomalies. The second branchial cleft cysts are the most common type; they account for up to 95% of the cases and can exist between the internal and external carotid arteries. The first cleft anomalies represent 1%-4%, and the third and fourth are uncommon. The third branchial cleft cysts course between the glossopharyngeal and hypoglossal nerves and connect to the pyriform sinus in the larynx. The fourth branchial cleft anomaly is rare and typically presents as a low anterior neck mass beneath the platysma and anterior to the sternocleidomastoid muscle. It usually passes deep into the common carotid and can loop around the aortic arch or the subclavian artery. It may also present with the skin opening near the medial lower border of the left sternocleidomastoid muscle. The exact course of the fourth cyst is not well characterized due to its rarity. Generally, these congenital defects manifest clinically until ages 10 to 40 as a firm mass or infected cyst, often associated with an upper respiratory tract infection that subsequently precipitates cyst enlargement [1,3,4].

Very few studies mention the presence of branchial cleft cysts in the anterior mediastinum, where the patients complain of respiratory issues, dysphagia, and chest pain. However, after an extensive literature review, only one case in posterior mediastinum was found [5]; with this case report, we are adding a new one.

## Case presentation

Our patient is a 54-year-old Hispanic woman with a long-standing history of obesity and well-controlled diabetes mellitus type 2. The patient also reported a 10-month history of asthma, characterized by shortness of breath that was exacerbated with exercise and associated with a dry cough. At that time, a chest radiograph was performed, which showed a 9.1 cm mass-like lesion in the posterior mediastinum. A couple of weeks later, a CT scan of the chest confirmed a left posterior mediastinal hypoattenuating mass measuring 6.1 x 6.8 x 5.9 cm at the level of the T10-T11 vertebral body.

The respiratory symptoms had greatly improved with albuterol, one to two monthly nebulizations, and montelukast. But then, the patient developed a new onset of lumbalgia three months after she was diagnosed with asthma. She described it as intense lower back pain and mild pain in the upper back; the lower back pain was so severe on two occasions that she had to visit the emergency department. On the first visit, when she started with lumbalgia, an MRI of the thoracic spine was performed. It showed mild degenerative changes and a well-circumscribed lesion within the left posterior mediastinum consistent with the one previously found. Five months later, the patient presented with the second episode of severe lower back pain and a visit to the emergency department. She underwent a CT scan of the abdomen and pelvis, which showed no abnormality other than the left posterior mediastinal mass, defined then as cystic.

She was then referred to the cardiothoracic surgery department two weeks later, still complaining of constant lower and upper back pain. The patient denied having any recent weight loss, chest pain, fever, cough, or shortness of breath, as well as any history of coronary artery disease, myocardial infarction, or renal disease. Cardiovascular and abdominal examinations were unremarkable.

**Figure 1:**
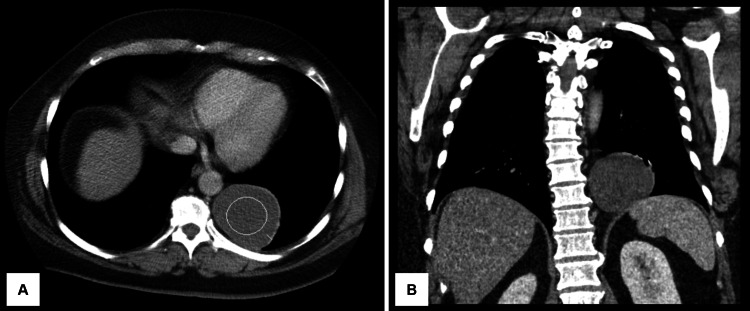
CT scan of the thorax A low-density mass (circled) is present in the left aspect of the posterior mediastinum, shown on the axial plane (A) and the coronal plane (B) of a CT scan of the chest. CT: computed tomography.

The following week she underwent a CT scan of the chest and thoracic spine with contrast. In the chest, a 5.6 x 6.3 x 6.1 cm low-density mass (30 Hounsfield units) in the left posterior mediastinum was found (Figures 1A, 1B). It did not appear to arise from the lung and was adjacent to, but separate from, the descending thoracic aorta. The lesion was not associated with significant pleural thickening or areas of pleural desiccation and did not involve the adjacent rib or thoracic spine vertebral bodies. In the thoracic spine, mild disc space narrowing and marginal osteophytosis were seen, with prominent osteophytes noted at T7-T8 and T8-T9.

Our patient was scheduled two weeks later for a robot-assisted thoracoscopic excision of the left posterior chest mass and possible open thoracotomy. All routine laboratory tests and electrocardiograms were within normal limits, except for the COVID-19 test, which turned out positive. The surgery was hence postponed for next month. In the follow-up visit to re-discuss the surgery, the patient mentioned that she had started with mild chest tightness on exertion and that the back pain had worsened. The patient entered the operating room the following day.

When dissecting along the left posterior chest lateral to the aorta, the patient was found to have a very large cystic structure, which was immediately adjacent to the pleura in the left chest. On aspiration, the cystic fluid was dark and thick and was sent for cultures and cytology. Then the cyst was excised using the robotic cautery and was sent to the pathology department, where a frozen section was performed; it showed a benign cyst wall with flattered epithelium without evidence of malignancy. After complete excision, the patient was brought to the surgical intensive care unit in stable condition.

**Figure 2:**
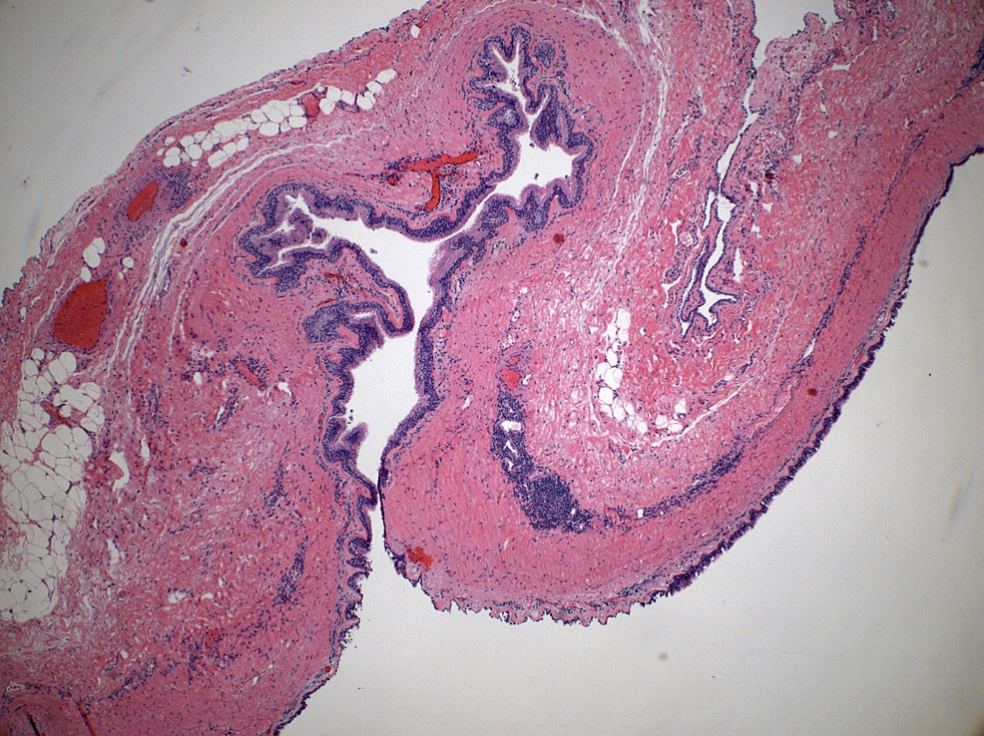
Panoramic picture of the branchial cleft cyst Mesothelium lining the external surface (superior aspect) and respiratory epithelium lining the inner surface (inferior aspect) cyst wall with lymphoid infiltrate (H&E 40x). H&E, hematoxylin and eosin.

**Figure 3:**
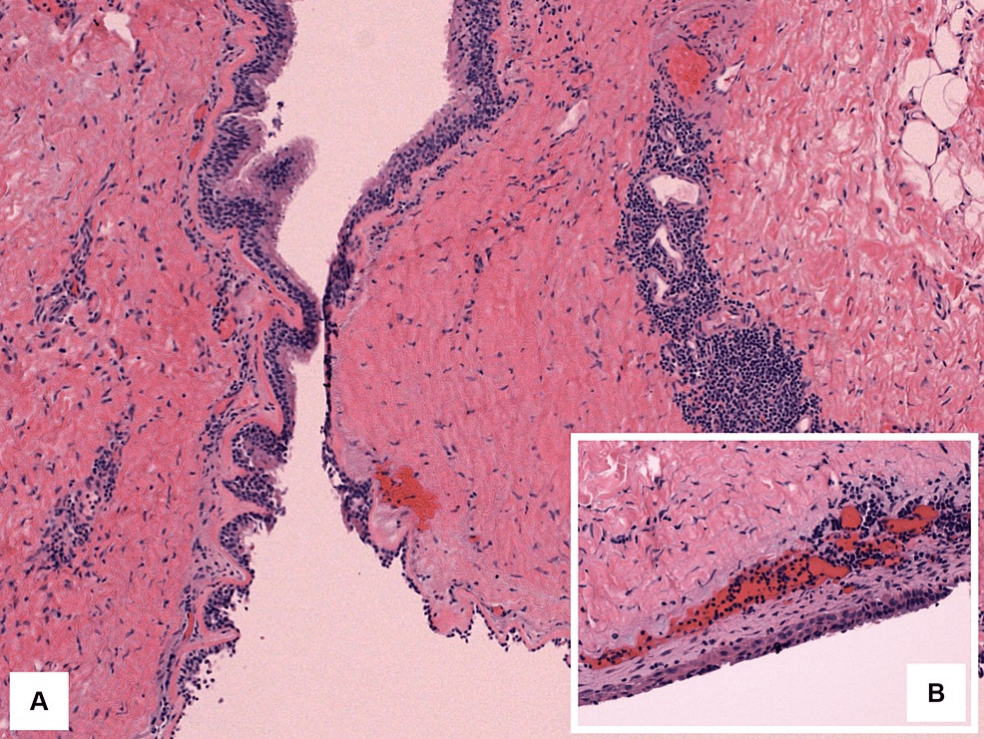
Branchial cleft cyst wall (A) Thin cyst wall with prominent lymphoid tissue (H&E, 100x), and (B) inset showing squamous metaplasia (H&E, 200x). H&E, hematoxylin and eosin.

The histopathology specimen was a membranous structure, with a cyst lining consisting of ciliated-type respiratory epithelium with patchy minimal squamous metaplasia and underlying smooth muscle/soft tissue stroma with benign lymphoid aggregates (Figures 2, 3A, 3B). The cytology specimen consisted of 5 ccs of thick brown fluid with moderate macrophages, with no evidence of atypia or malignancy. The final diagnosis was a branchial cleft cyst.

The postoperative course was uneventful, the cyst fluid cultures showed no growth at seven days, and our patient was discharged on the 10th day of hospitalization with no complications. At a follow-up visit two months after the surgery, the patient reported complete remission of lumbalgia and clinically significant improvement regarding respiratory symptoms.

## Discussion

The mediastinum is the central compartment in the thorax surrounded by the two pleural sacs. There are the superior and the inferior mediastina; the latter further divides into the anterior, middle, and posterior mediastina. The posterior mediastinum borders are the T4 vertebra, the T12 vertebra, the posterior pericardium, and the spine. It contains the descending thoracic aorta, azygos veins, thoracic duct, esophagus, esophageal plexus, vagus nerve, and lymphatic trunks. It has great significance when diagnosing suspected masses, and a neurogenic tumor is the most common diagnosis of a posterior mediastinal mass, followed by the bronchogenic cyst, enteric cyst, xanthogranuloma, diaphragmatic hernia, meningocele, and paravertebral abscess [6,7].

Neurogenic tumors derive from the tissue of the neural crest; they include schwannomas, neurofibromas, ganglioneuromas, and neuroblastomas. Seventy percent to 80% are benign, and nearly half are asymptomatic and discovered incidentally. Clinical presentation varies depending on size and location; they may cause thoracic pain, radicular pain, dorsal neuralgia, cough, dyspnea, or weight loss.

Bronchogenic cysts are also commonly found in the posterior mediastinum. They are developmental anomalies that occur during embryogenesis as an abnormal branching of the foregut or tracheobronchial tree. They usually arise posterior to the carina or main stem bronchus and may communicate with the tracheobronchial tree. These masses are often asymptomatic, but some adults can present with respiratory symptoms, chest pain, or dysphagia. Over half of all bronchogenic cysts are found incidentally [6].

Although rare, one case of a posterior mediastinal branchial cleft cyst has been reported before [5]. Branchial cleft cysts are congenital anomalies that, because of the specific trajectories of pharyngeal arch remnants during embryonic development, are usually present as a mass in the cervical area. They are well-enclosed lesions without any communication with surrounding tissues [2,8,9]. The lower cervical area is expected to be affected in the second and third branchial cleft anomalies. The fourth cleft cyst would generally appear in the lateral lower third of the neck. The classic clinical presentation makes sense with their most common topographic location, and it includes acute suppurative thyroiditis, recurrent cervical abscess, and upper respiratory symptoms [10,11]. The anterior and superior mediastina are unusual sites for this entity but appeared in five case reports with symptoms such as wheezing, dyspnea, cough, and chest pain [2,12-15].

In 10 months, our patient presented shortness of breath that was exacerbated with exercise, dry cough, severe lumbalgia in the lower and upper back, and chest tightness. The left posterior mediastinal mass at T10-T11 was thought to be representing a neurogenic tumor. Other differential diagnoses included foregut duplication cyst, solitary fibrous tumor of the pleura, and spindle cell neoplasm. It was not until the complete surgical excision of the mass that the final diagnosis of a branchial cleft cyst was made. This was due to the histopathological findings of a cyst lining consisting of ciliated-type respiratory epithelium with patchy minimal squamous metaplasia and an underlying smooth muscle/soft tissue stroma with benign lymphoid aggregates.

The classic histopathology description of a branchial cleft cyst is a thin wall composed of either stratified squamous epithelium or columnar ciliated epithelium that typically shows lymphoid tissue. Among other masses that arise in the posterior mediastinum, histologically, bronchogenic cysts are the principal differential diagnosis since branchial cleft cysts sometimes present smooth muscle, mucous glands, and cartilage, although bronchogenic cysts are not associated with lymphoid tissue [10].

A pure mediastinal presentation for a branchial cleft cyst is so rare that its possibility has been debated before. Downey and Ward originally postulated that for a branchial cleft cyst in that site to occur, it would necessarily be of fourth branchial cleft origin [2,12,16]. The trajectory of the fourth branchial arch development follows a long path that starts by the caudal end of the pyriform sinus, then between the trachea and carotid vessels, deep into the clavicle, loops below the aortic arch into the left side and the mediastinum. A fistula would continue the path toward the lateral cervical area. However, the long and tortuous pathway of the fourth branchial development might be an explanation for why a complete fistula has not been reported in the literature [11].

Our patient’s initial respiratory problems could have been part of an early clinical presentation of her branchial cleft cyst; however, because they were controlled with albuterol, nebulizations, and montelukast, the diagnosis of asthma was favored. The description of the lumbalgia also corresponded to the location of the mass, and it is reasonable for a neurogenic tumor to have been considered a differential diagnosis; the osteophytosis at T7-T8 and T8-T9 could have also been one cause. It is a new finding that has not been mentioned in previous cases of branchial cleft cysts. Around the time when she was diagnosed with COVID-19, the patient developed chest tightness, but due to the absence of other symptoms or imaging findings, the chest tightness was, most probably, a progression of the branchial cleft cyst.

At a follow-up visit two months after the surgery, the patient mentioned complete remission of lumbalgia and clinically significant improvement concerning respiratory symptoms. According to the literature and as seen in our patient, the treatment for branchial cleft cysts is complete surgical excision, as the symptoms resolve and the probability of recurrence is minimal. Open thoracotomy is a technique that has been used because it permits the surgeons to access the cysts and surrounding tissues while having more visibility and a broad area to intervene if there is a complication. On the other hand, the robot-assisted thoracoscopic excision offers better maneuverability, resulting in the complete excision of masses located in difficult sites without damaging the adjacent structures and therefore experiencing fewer complications. To the best of our knowledge, there has only been one case of a branchial cleft cyst in the posterior mediastinum [5]. It was diagnosed in a two-year-old Kenyan girl with a history of recurrent chest infections and dysphagia, different from our 54-year-old patient who presented with respiratory symptoms and lumbalgia, and as well as the previous case, she had a complete recovery after surgical removal.

## Conclusions

Branchial cleft cyst represents the most common diagnosis for a congenital lateral neck mass in pediatrics, and only one case in the posterior mediastinum has been described. This is a case of a 54-year-old Hispanic woman with a recent onset of shortness of breath that was exacerbated with exercise, dry cough, and lumbalgia, which symptoms we associate with the presence of a left posterior mediastinum mass diagnosed as a branchial cleft cyst. The symptomatology, as well as the origin and mechanism of the extension of a branchial cleft cyst to the posterior mediastinum, remains unknown; we conclude that it could have only originated from the fourth branchial cleft. The diagnosis relies on the histopathological examination; therefore, it should be considered after ruling out the most common differential diagnosis of mediastinal lesions for prompt management, which we recommend to be robot-assisted thoracoscopic excision for complete symptom remission and a safer approach.

## References

[1] Waldhausen JH (2006). Branchial cleft and arch anomalies in children. Semin Pediatr Surg.

[2] Park SH, Kim SH, Shin HW, Jo HC, Son MY, Gong JH (2008). The occurrence of a branchial cleft cyst in the anterior mediastinum: a case report. J Korean Soc Radiol.

[3] Choi SS, Zalzal GH (1995). Branchial anomalies: a review of 52 cases. Laryngoscope.

[4] Coste AH, Lofgren DH, Shermetaro C (2022). Branchial cleft cyst. StatPearls [Internet].

[5] Rashid A, Ahmad V, Qazi S, Billoo AG, Rashid S, Saleem AF (2014). Posterior mediastinal branchial cleft cyst: an unusual site. J Coll Physicians Surg Pak.

[6] Duwe BV, Sterman DH, Musani AI (2005). Tumors of the mediastinum. Chest.

[7] Brunicardi FC, Andersen DK, Billiar TR (2014). Schwartz's Principles of Surgery.

[8] Prosser JD, Myer CM 3rd (2015). Branchial cleft anomalies and thymic cysts. Otolaryngol Clin North Am.

[9] Ozolek JA (2009). Selective pathologies of the head and neck in children: a developmental perspective. Adv Anat Pathol.

[10] Shugar MA, Healy GB (1980). The fourth branchial cleft anomaly. Head Neck Surg.

[11] Hwang TZ, Lin YJ, Tsai ST (2000). Fourth branchial cyst presenting with neonatal respiratory distress. Ann Otol Rhinol Laryngol.

[12] Downey WL, Ward PH (1969). Branchial cleft cysts in the mediastinum. Arch Otolaryngol.

[13] Kotecha V, Muturi A, Ruturi J (2015). Branchial cysts: an unusual cause of a mediastinal mass: a case report. J Med Case Rep.

[14] Murdoch MJ, Culham JA, Stringer DA (1995). Pediatric case of the day. Infected fourth branchial pouch sinus with an extensive complicating cervical and mediastinal abscess and left-sided empyema. Radiographics.

[15] Lewis C, Lewis M, Vaughan R (2006). Branchial cyst: a case of unusual retrosternal extension into the anterior mediastinum. Internet J Thorac Cardiovasc Surg.

[16] Meng F, Zhu Z, Ord RA, Zhang T (2019). A unique location of branchial cleft cyst: case report and review of the literature. Int J Oral Maxillofac Surg.

